# Genome-wide identification of dysregulated alternative splicing and RNA-binding proteins involved in atopic dermatitis

**DOI:** 10.3389/fgene.2024.1287111

**Published:** 2024-03-01

**Authors:** Yaqi Yang, Hao Chen, Qing Jiang, Lin Yang, Rongfei Zhu, Nan Huang

**Affiliations:** Department of Allergy, Tongji Hospital, Tongji Medical College of Huazhong University of Science and Technology, Wuhan, China

**Keywords:** atopic dermatitis, RNA-binding proteins, alternative splicing, immune and inflammatory response, genome-wide analysis

## Abstract

**Objectives:** We explored the role and molecular mechanisms of RNA-binding proteins (RBPs) and their regulated alternative splicing events (RASEs) in the pathogenesis of atopic dermatitis (AD).

**Methods:** We downloaded RNA-seq data (GSE121212) from 10 healthy control skin samples (healthy, Ctrl), 10 non-lesional skin samples with AD damage (non-lesional, NL), and 10 lesional skin samples with AD damage (lesional, LS). We performed the analysis of differentially expressed genes (DEGs), differentially expressed RBPs (DE-RBPs), alternative splicing (AS), functional enrichment, the co-expression of RBPs and RASEs, and quantitative polymerase chain reaction (qPCR).

**Results:** We identified 60 DE-RBP genes by intersecting 2141 RBP genes from existing reports with overall 2697 DEGs. Most of the DE-RBP genes were found to be upregulated in the AD LS group and related to immune and apoptosis pathways. We observed different ASEs and RASEs among the healthy, AD NL, and AD LS groups. In particular, alt3p and alt5p were the main ASEs and RASEs in AD NL and AD LS groups, compared to the healthy group. Furthermore, we constructed co-expression networks of DE-RBPs and RAS, with particular enrichment in biological pathways including cytoskeleton organization, inflammation, and immunity. Subsequently, we selected seven genes that are commonly present in these three pathways to assess their expression levels in the peripheral blood mononuclear cells (PBMCs) from both healthy individuals and AD patients. The results demonstrated the upregulation of four genes (IFI16, S100A9, PKM, and ENO1) in the PBMCs of AD patients, which is highly consistent with DE-RBP genes analysis. Finally, we selected four RAS genes regulated by RBPs that were related to immune pathways and examined their RASEs in PBMCs from both AD patients and healthy controls. The results revealed an increased percentage of RASEs in the *DDX60* gene in AD, which is highly consistent with AS analysis.

**Conclusion:** Dysregulated RBPs and their associated RASEs may have a significant regulatory role in the development of AD and could be potential therapeutic targets in the future.

## Introduction

Atopic dermatitis (AD) is a refractory, recurrent, and systemic type 2 inflammatory dermatosis that affects approximately 20% of children and 10% of adults worldwide, with substantial variation between countries; however, the incidence of AD is steadily increasing globally (Bylund et al., 2020; Sroka-Tomaszewska and Trzeciak, 2021). The chronic, relapsing course of AD and its complications, economic burden, and the whole family’s involvement in the treatment process significantly impact the quality of life for both patients and their families (Ali et al., 2020). The primary therapeutic goal for AD is to achieve early intervention and sustained control, leading to remission (Wollenberg et al., 2022). Therefore, there is an urgent need for further research into the mechanisms of AD development, aiming to develop effective preventive strategies and enhance treatment options.

The pathophysiology of AD is complex and multifactorial. Currently, AD is known to be influenced by genetic disorders, environmental factors, epidermal barrier disruption, skin microbiota dysbiosis, and altered immune response (Sroka-Tomaszewska and Trzeciak, 2021). Complex changes in innate and adaptive immunity at the genetic level contribute to the diverse phenotypes and endotypes observed in AD (Tokura and Hayano, 2022). Extrinsic AD, characterized by skin barrier damage and a high prevalence of filaggrin mutations, is associated with an increased likelihood of protein allergy and food sensitivities (Palmer et al., 2006; Astolfi et al., 2021), while the endotype is defined as the molecular mechanisms. The molecular pathology of AD involves aberrant immune activation and interactions among the skin, immune, and neuronal cells (Kwatra et al., 2022). The endotype repertoire includes the activation of type 1 and type 2 cytokines, as well as IL-17/IL-22. It also shows impairment of the epidermal barrier and abnormalities in intercellular lipids (Tokura and Hayano, 2022). However, the AD occurrence and development mechanism is unclear, and further research is necessary.

Regulation of gene expression at the RNA level is crucial for various biological processes. The RNA-binding proteins (RBPs) can recognize cis-elements or specific structures in the 5′-untranslated region (UTR), 3′-UTR, or intron of the mRNA and regulate essentially every event in the lifetime of an RNA molecule, including RNA splicing, capping, polyadenylation, export, localization, phosphorylation, and decay (Akira and Maeda, 2021; He et al., 2023). Dysregulation of RBPs can lead to cellular dysfunction and contribute to infections, cancers, autoimmune diseases, and genetic diseases, (Yoshinaga and Takeuchi, 2019; Gebauer et al., 2021; Hashimoto and Kishimoto, 2022). The tristetraprolin (TTP) family is made up of a few RBPs that bind to specific AU-rich sites within the 3′-UTRs of certain mRNAs, which helps promote mRNA deadenylation and decay. In mice that lack TTP, there is a severe inflammatory syndrome that includes arthritis, myeloid hyperplasia, dermatitis, autoimmunity, and cachexia, which is caused, in part, by an excessive accumulation of proinflammatory chemokine and cytokine mRNAs and their encoded proteins (Snyder and Blackshear, 2022). Research has shown that dermal fibroblasts in psoriatic skin lesions play a significant role in producing inflammatory mediators such as IL-6, CXCL8, and CXCL2. The production of these cytokines is regulated by ZFP36 family members, including ZFP36, ZFP36L1, and ZFP36L2, which are RBPs that possess mRNA-degrading properties, (Angiolilli et al., 2022). However, there is limited information regarding the expression pattern and abnormal regulation of RBPs in AD.

Alternative splicing (AS) is a driving factor for post-transcriptional regulation, which arranges the introns and exons of immature pre-mRNA in various ways to produce different transcripts. Networks of functionally coordinated and biologically important AS events (ASEs) are continuously being discovered in various physiological and pathological states (Ule and Blencowe, 2019). Some studies demonstrated that abnormal RNA splicing contributes to inflammatory skin disorders in AD (Wolf et al., 2007; Rehman et al., 2019; Moldovan et al., 2021; Ni et al., 2022). Research has investigated the impact of different AS forms of IL-33 on inflammation in asthma and AD, revealing a close association between one of these forms and the occurrence and development of type 2 inflammation (Gordon et al., 2016). Some studies have examined the expression of AS forms of IL31 in AD and their influence on inflammatory responses (Zhang et al., 2008; Craig et al., 2021). These findings indicate that AS genes may serve as promising molecular targets for AD. Nonetheless, there is a limited amount of research on the role of AS and its regulatory mechanism in AD.

It is hypothesized that during the development of AD, there may be abnormal expression and ASEs in numerous genes, with some of these genes participating in crucial functional pathways that influence the progression of atopic diseases. RBPs, as crucial regulators of AS, are likely to play an important role in its regulation. This study used RNA-seq data (GSE121212) obtained from 10 healthy control skin samples (healthy, Ctrl), 10 non-lesional skin samples with AD damage (non-lesional, NL), and 10 lesional skin samples with AD damage (lesional, LS) (Tsoi et al., 2019). Subsequently, we conducted an analysis of differentially expressed RBPs (DE-RBPs) and regulated alternative splicing events (RASEs) in the skin of healthy individuals and those with AD. Furthermore, we established a co-variation network between DE-RBPs and RASEs. The study findings revealed the potential function of RBP regulation of AS in the development of AD. Finally, we analyzed the expression of selected DE-RBPs and RASEs in our cohort of clinically healthy individuals and AD patients. Further investigation into these DE-RBPs could aid in predicting the onset of AD and reveal the underlying mechanisms of RBPs.

## Materials and methods

### Retrieval and process of public data

We collected and analyzed RNA-seq data (GSE121212) from 10 healthy control skin samples, 10 non-lesional skin with AD damage, and 10 lesional skin samples with AD damage. Demographic data are shown in Supplementary Table S1. Public sequence data files were downloaded from the Sequence Read Archive (SRA) (https://www.ncbi.nlm.nih.gov/geo/query/acc.cgi?acc=GSE121212). The SRA Run files were converted to fastq format using the NCBI SRA tool fastq-dump. We used the FASTX-Toolkit to trim low-quality bases from the raw reads (v.0.0.13; http://hannonlab.cshl.edu/fastx_toolkit/) (Salzberg et al., 2010). Finally, the clean reads underwent evaluation through FastQC (http://www.bioinformatics.babraham.ac.uk/projects/fastqc).

### Reads alignment and differentially expressed gene analysis

Clean reads were aligned to the human GRCh38 genome via HISAT2 (v.2.2.1) (Kim et al., 2015). Uniquely mapped reads were screened for further analyses. We, then, calculated the reads number located on each gene. The expression levels of genes were evaluated using fragments per kilobase of exon per million fragments mapped (FKPM). DESeq2 (v.1.30.1) software (https://bioconductor.org/packages/release/bioc/html/DESeq2.html) was used to perform differential gene expression analysis using the reads count file and analyze the differential expression between two or more samples and, thus, determine whether a gene was differentially expressed by calculating the fold change (FC) and false discovery rate (FDR) (Love et al., 2014).

**There are two important parameters**

1) FC: Fold change, the absolute ratio of expression change
2) FDR: False discovery rate

**The criteria of significant difference expression were as follows**

FC≥2 or≤0.5,FDR≤0.05.

### Identification of differentially expressed RBPs in the groups

Differentially expressed genes (DEGs) were screened using the DESeq2 software on the raw count data. To determine if a gene was expressed differently, we analyzed the results using the fold change criteria (FC ≥ 2 or ≤ 0.5) and false discovery rate (FDR ≤ 0.05). The expression profile of differentially expressed RBPs was filtered out from all DEGs using a catalog of 2,141 RBPs retrieved from four previous reports (Castello et al., 2012; Gerstberger et al., 2014; Castello et al., 2016; Hentze et al., 2018).

### Alternative splicing analysis

The splicing site usage variation analysis (SUVA) (v2.0) pipeline was used to define and quantify ASEs and RASEs (Cheng et al., 2021). The different splicing of each group was analyzed. Reads proportions of the SUVA AS event (pSAR) of each ASE were calculated. SUVA operates based on five distinct ASE models, which are determined by changes in splice site usage. These models differentiate between a shared and an alternative splice site (alt3p; alt5p), both of which can be optional (olp, contains). SUVA uses either both front splice sites or none at all (ir).

The ASEs and RASEs between the samples were defined and quantified using the ABLas pipeline, as described previously (Xia et al., 2017). The ABLas detection of 10 types of ASEs was based on the splice junction reads, including exon skipping (ES), alternative 5′splice site (A5SS), alternative 3’splice site (A3SS), intron retention (IR), mutually exclusive exons (MXEs), mutually exclusive 5′-UTRs (5pMXE), mutually exclusive 3′-UTRs (3pMXE), cassette exon, A3SS&ES, and A5SS&ES. We used Fisher’s exact test to determine the statistical significance of our data. Specifically, we input the alternative reads and model reads of our samples. To measure the difference in alternatively spliced reads and constitutively spliced reads between the compared samples, we calculated the RASE ratio. We set the threshold for RASE detection at an RASE ratio of ≥0.2 and a *p*-value of ≤0.05. For repeated comparisons, we used Student’s t-test to evaluate the significance of the ratio alteration of ASEs. Any events that were significant at a *p*-value cutoff of 0.05 were considered non-intron retention (NIR) RASEs.

### Co-expression analysis

We conducted a co-expression analysis of RBP and RAS (pSAR≥50%). Additionally, we calculated the Pearson correlation coefficient between the FPKM value of RBP and the ratio value of RAS and filtered for RBP and RAS relationship pairs with an absolute value of correlation coefficient ≥0.6 and a *p*-value ≤0.01.

### Functional enrichment analysis

Gene Ontology (GO) terms and KEGG pathways were identified using KOBAS 2.0 (http://kobas.cbi.pku.edu.cn/) (Xie et al., 2011). To determine the enrichment of each term, we used the hypergeometric test and the Benjamini–Hochberg FDR controlling procedure.

### Participants

Samples from 15 April to 15 June 2023 were searched in the peripheral blood mononuclear cell (PBMC) database of our department, and 15 AD patients and 6 healthy control cases were found. The following were the inclusion criteria for the study group: 1) an adult patient was diagnosed with atopic dermatitis and without other skin-related diseases; 2) patients had received only traditional topical and oral anti-allergy medications, without systemic oral glucocorticoids, immunosuppressants, or targeted therapies. The control group’s inclusion criterion was serum-specific immunoglobulin E (sIgE) testing excludes subjects with allergic diseases, no skin diseases, and no immune system disorders. Demographic data are shown in Supplementary Table S2.

### Quantitative polymerase chain reaction

Total RNA was isolated using the TRIzol reagent (Invitrogen, 15596026) from PBMCs which were stored at −80°C. The RNA (2 ug) was then reverse transcribed, following the manufacturer’s instruction from Illumina^®^ (SeqHealth Co., Ltd., DR08042). Fluorescence quantitative polymerase chain reaction (qPCR) with SYBR Green I fluorescent dye (Vazyme Biotech, Q111-02/03) was used to measure gene transcription levels, following the manufacturer’s recommendations. The results were standardized to endogenous *GAPDH* as an internal control. Primers used for qPCR are listed in Supplementary Table S3.

### Other statistical analysis

Principal component analysis (PCA) was performed using R package factoextra (v 1.0.7) (https://cloud.r-project.org/package=factoextra) to show the clustering of samples with the first two components. The samples’ gene reads were normalized by FPKM before using an in-house script (called Sogen) to visualize next-generation sequence data and genomic annotations. The pheatmap package (v 1.0.12) (https://cran.r-project.org/web/packages/pheatmap/index.html) in R was used to perform the clustering based on the Euclidean distance. All datasets are presented as the mean ± SEM. Student’s t-test was used for comparisons between the two groups. *p* < 0.05 was considered significant.

## Results

### Part 1: Transcriptome analysis of DE-RBPs in LS and NL samples with AD compared with healthy samples

**FIGURE 1:**
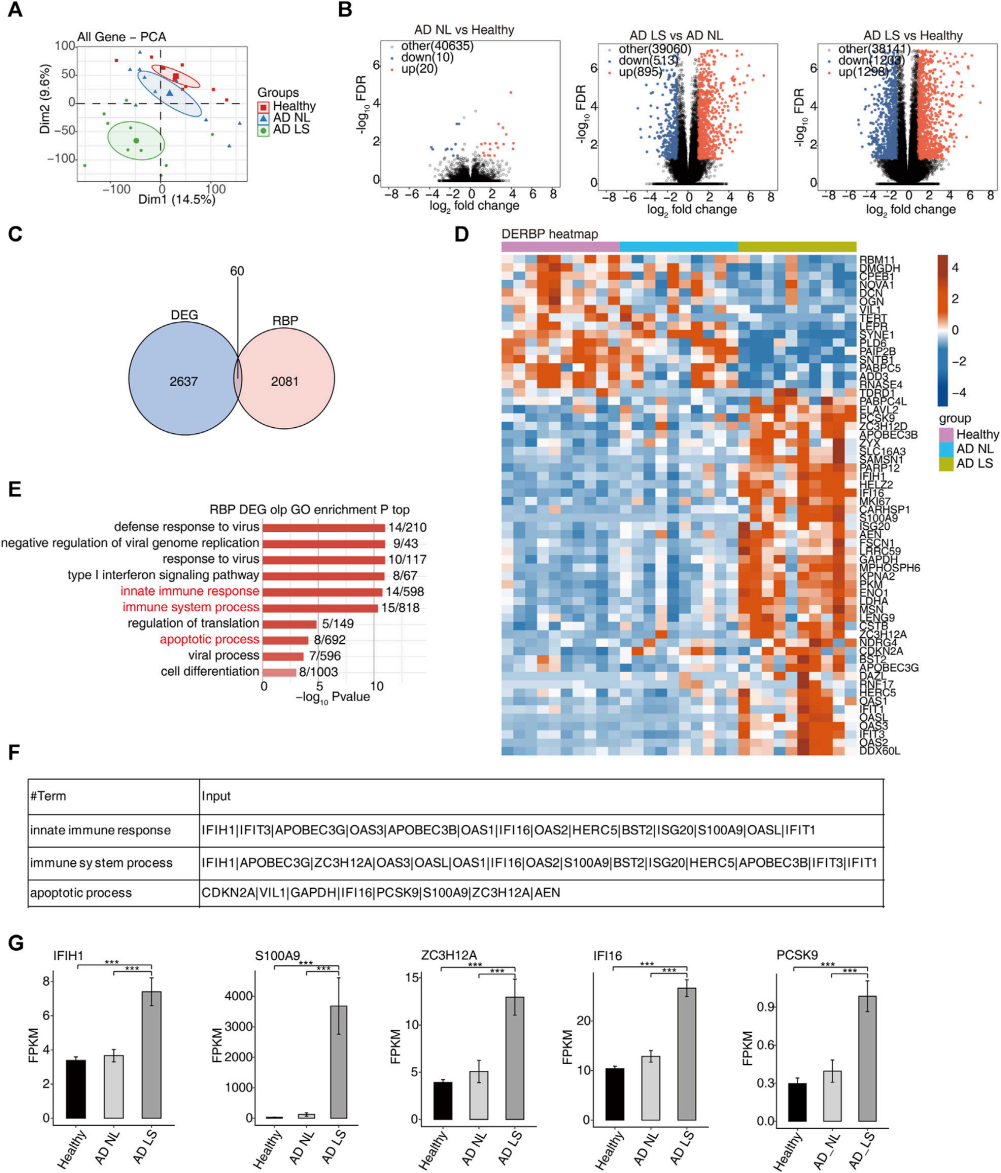
Transcriptome analysis of DE-RBPs in atopic dermatitis lesional and atopic dermatitis non-lesional samples compared with healthy samples. **(A)** PCA based on the FPKM value of all detected genes. The ellipse for each group is the confidence ellipse. **(B)** Volcano plots presenting all DEGs in the AD_LS vs. healthy, AD_NL vs. healthy, and AD_LS vs. AD_NL comparison groups. FDR ≤0.05 and FC ≥ 2 or ≤0.5. **(C)** Venn diagram showing the overlap of DEGs and RBPs. **(D)** Heatmap diagram showing the expression profile of specific DE-RBPs in the three comparison groups. **(E)** Bar plot showing the most enriched GO biological process results of specific DE-RBPs in the three comparison groups. The color scale shows the row-scaled significance (-log10-corrected *p*-value) of the terms. **(F)** Sheet showing the genes enriched in the key GO pathway. **(G)** Bar plot showing the expression pattern and statistical difference of DE-RBPs. Error bars represent mean ± SEM. ***p-value <0.001.

To gain a comprehensive understanding of the disease-related RBP regulators in AD patients, first, we collected and analyzed RNA-seq data (GSE121212) from 10 healthy samples, 10 NL samples, and 10 LS samples with AD. The PCA showed significant differences in the transcriptomic genes between the AD LS group and AD NL group, as well as between the AD LS group and healthy group. However, there was minimal difference observed between the AD NL group and healthy group (Figure 1A). A total of 1298 upregulated DEGs and 1203 downregulated DEGs were identified between the healthy group and AD LS group, while 895 upregulated DEGs and 513 downregulated DEGs were observed between the AD NL group and AD LS group. However, only 20 upregulated DEGs and 10 downregulated DEGs were found between the healthy group and AD NL group (Figure 1B). This suggests these gene expressions led to significantly different transcriptional characteristics in AD development.

Second, we collected 2141 RBP genes from existing reports and intersected with the overall 2697 DEGs, resulting in the identification of 60 DE-RBP genes (Figure 1C). The heatmap showed that more DE-RBP genes (Figure 1D) and DEGs (Supplementary Figure S1A) were upregulated in the AD LS group. These findings suggest that the excessive activation of RBP genes during the course of AD development may contribute to the progression of the illness.

In order to gain insights into the function of these DE-RBP genes, we performed a GO enrichment analysis. Figure 1E shows the top 10 most enrichment biological process results of DE-RBPs in the three groups; we mainly focused on immune and apoptosis pathways, including the innate immune response, immune system process, and apoptotic process. Previous studies have demonstrated that the dysfunction of T cells and apoptosis of keratinocytes contribute to the occurrence and progression of AD (Akdis et al., 2000; Yang et al., 2020; Das et al., 2022), aligning with the findings of the above analysis. It is implied that RBPs may influence the onset and progression of AD by regulating immunity and apoptosis. Additionally, Figure 1F illustrates the DE-RBP genes related to immune and apoptosis pathways. Furthermore, Figure 1G depicts the expression pattern and statistical differences of key DE-RBP genes, including IFIH1, S100A9, ZC3H12A, IFI16, and PCSK9. The expression levels of these five DE-RBP genes were significantly higher in the AD LS group than in both the healthy group and AD NL group. For IFIH1: CADM140, a 140-kD polypeptide, binds to IFIH1, a cytosolic helicase receptor for viral RNA and a mediator of innate immune responses. Upregulation of CADM140 has been observed in dermatomyositis and related autoimmune diseases (Zahn et al., 2011). For ZC3H12A: ZC3H12A encodes the RNase monocyte chemotactic protein-1-induced protein-1 (MCPIP1), an endoribonuclease that destabilizes inflammatory cytokine mRNAs. It is upregulated by IL-17A (Ruiz-Romeu et al., 2016). For PCSK9: PCSK9 has been reported to be associated with inflammation in AD (Valenzuela et al., 2020). These findings suggest that these RBP genes may play regulatory roles in immune- and apoptosis-related processes during the development of AD.

We, then, analyzed the specific DEGs of the healthy group, AD NL group, and AD LS group to investigate the differences in their skin injury mechanisms. The GO analysis revealed that the upregulated genes in both the AD NL group and AD LS group, as well as the upregulated genes in the AD LS group and healthy group, were mainly enriched in immune and signaling pathways, including the cytokine- or chemokine-mediated signaling pathway, immune system process, innate immune response, and immune response (Supplementary Figure S1B and Supplementary Figure S1E). These pathways are implicated in immune inflammation during the progression of AD. The downregulated genes were mainly enriched in biological pathways related to multicellular organism development and transport, including ion transport, chemical synaptic transmission, and ion transmembrane transport (Supplementary Figure S1C and Supplementary Figure S1F). These downregulated pathways may create an environment conductive to immune response. In the GO analysis of the AD NL group and healthy group, the most upregulated genes were enriched in biological pathways such as epidermis development, keratinization, cornification, and keratinocyte differentiation (Supplementary Figure S1D). These pathways are implicated in the maintenance and repair of the skin barrier.

### Part 2: Numerous alternative splicing events related to inflammatory and immune responses were identified during atopic dermatitis development

**FIGURE 2:**
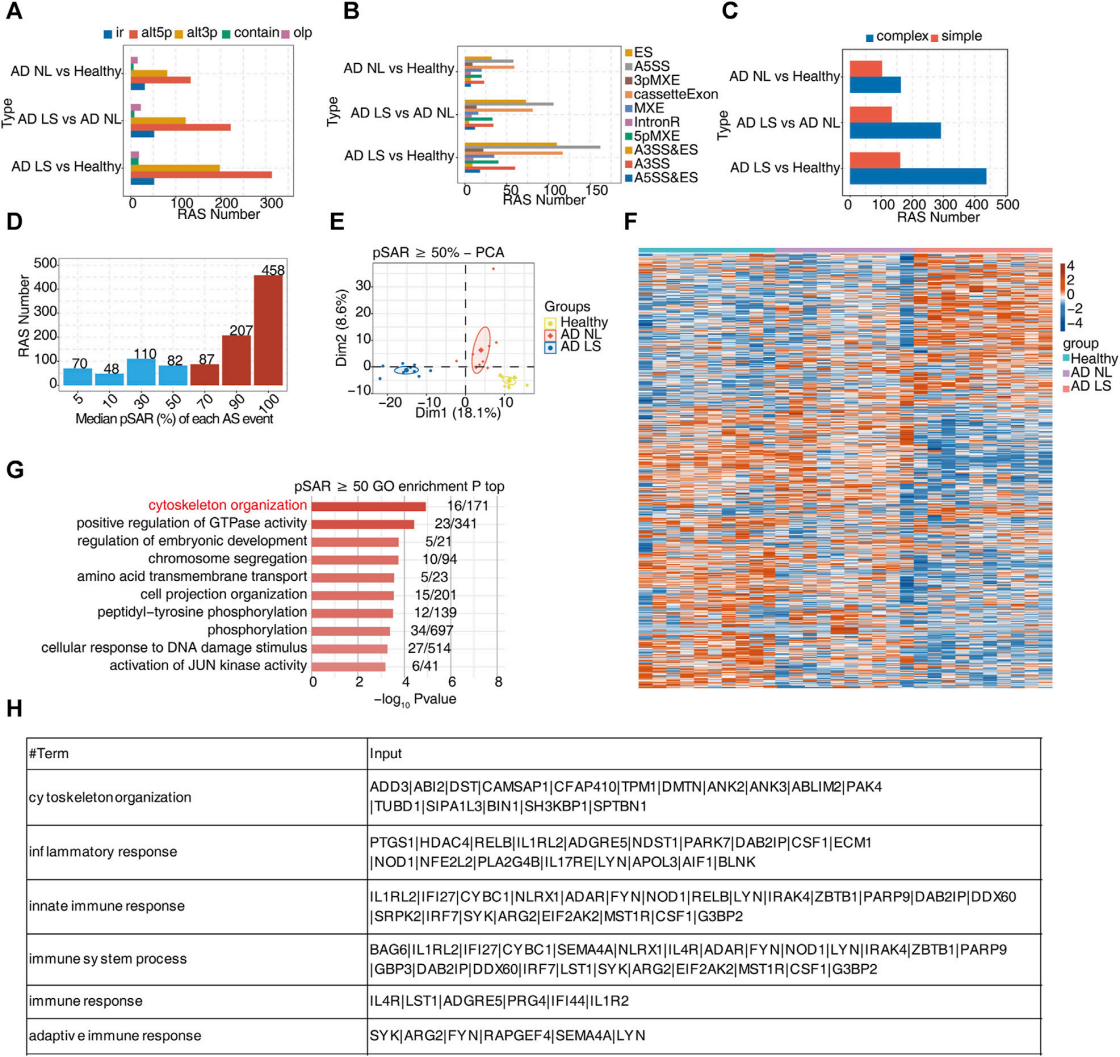
A large number of alternative splicing events related to inflammatory and immune responses were identified in atopic dermatitis development. **(A)** Bar plot showing several regulatory RAS detected using the SUVA in each group. **(B)** Splice junction constituting RAS events detected using the SUVA was annotated to classical AS event types. The number of each classical AS event type is shown with a bar plot. **(C)** Complex and simple variable splicing event detection cases. **(D)** Bar plot showing RAS with different pSAR values. RAS whose pSAR (Reads proportion of SUVA AS event) ≥ 50% were labeled. **(E)** PCA based on RAS with pSAR ≥50%. The ellipse for each group is the confidence ellipse. **(F)** Heatmap showing the splicing ratio of specific RAS (pSAR ≥50%) in the three groups. **(G)** Bar plot exhibiting the most enriched GO biological process results of specific RAS (pSAR ≥50%) genes in the three groups. The color scale shows the row-scaled significance (-log10 corrected *p*-value) of the terms. **(H)** Sheet exhibiting several key GO biological process results of RAS (pSAR ≥50%).

For the analysis of transcript levels in healthy and AD patients, we still do not know the mechanism of the difference. To further study the post-transcriptional levels of DEGs and characterize AS regulation in AD, we analyzed the RNA-seq data using the software SUVA. Unlike most current AS analysis tools unable to analyze complex splices, SUVA can effectively break down complex splicing events into five types of splicing connection pairs. By analyzing both real and simulated data, it has been demonstrated that SUVA exhibits greater sensitivity and accuracy in detecting AS events compared to other methods (Cheng et al., 2021). Our analysis revealed the presence of distinct ASEs and RASEs among the healthy group, AD NL group, and AD LS group. Specifically, alt3p and alt5p were the main ASEs and RASEs in the AD NL group and AD LS group, relative to the healthy group (Figure 2A; Supplementary Figure S2A). Corresponding SUVA-identified ASEs to the classical ASEs, the results showed the most common ASEs and RASEs in humans belonged to the cassette exon, A3SS, and A5SS in the AD NL group and AD LS group, compared to the healthy group. Additionally, we observed a higher number of AS types and RAS types in the skin of AD patients compared to healthy individuals (Figure 2B; Supplementary Figure S2B). Figure 2C shows that complex splicing was the main type among the RASEs. Based on our findings, we hypothesized that the RASE genes play a crucial role in the mechanism of skin injury in AD.

Due to a splicing event involving two transcripts, which may account for a very small proportion of the entire gene expression, we aimed to identify the more dominant transcripts in splicing events. Specifically, we counted the number of splicing events with different proportions of RASEs in the region covered by all reads. Additionally, we removed a portion of splicing events that only accounted for a small proportion (pSAR<50%). We selected a total of 752 events with pSAR≥50% for subsequent analyses (Figure 2D). Next, we performed PCA using the pSAR values of the differential RASEs presented in the samples. The result showed significant separation among the three groups, suggesting a close association between post-transcriptional RASE genes and the development of AD (Figure 2E). To analyze these RASE features more intuitively, we performed cluster analysis on the samples using the pSAR values of the different RASEs in each sample. The results were consistent with observed transcriptional differences. Specifically, the splicing ratio of RAS in the AD LS group was significantly different from that in both the healthy group and the AD NL group, indicating significant differences in the genes involved in differential RASEs between the AD LS group and the healthy and AD NL groups. However, no significant difference was observed between the healthy group and the AD NL group (Figure 2F).

To analyze the function of these differential RASEs, we performed GO and KEGG functional enrichment analyses on these genes undergoing RASEs, and the pictures showed the top 10 enriched GO biological process (Figure 2G) and KEGG terms (Supplementary Figure S2C). We mainly focus on the biological pathways such as cytoskeleton organization, inflammation, and immunity, which are shown in the table (Figure 2H). The RASEs of these genes associated with inflammation and immunity may influence the onset and development of AD.

### Part 3: Construction of a co-dysregulated network between RBPs and AD-associated RAS in AD

**FIGURE 3:**
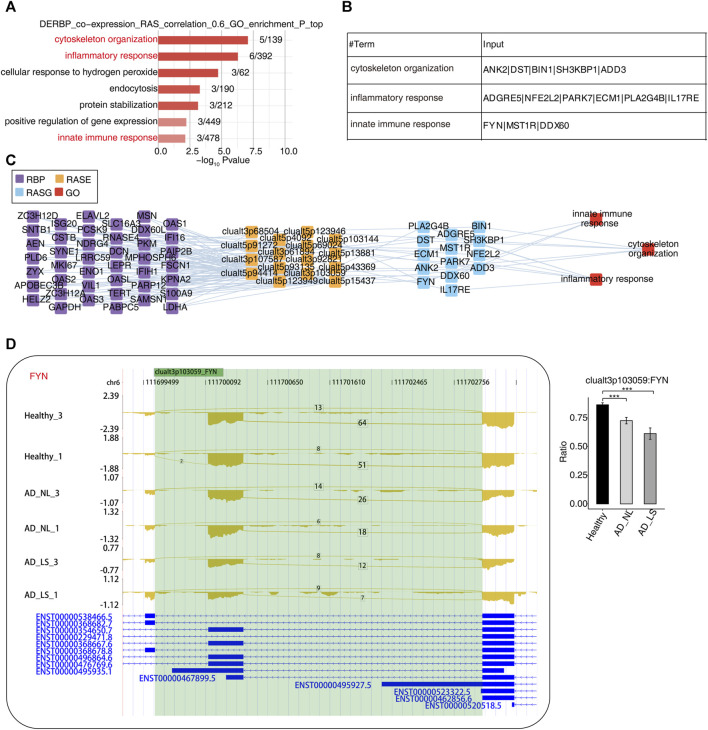
Construction of a co-dysregulated network between RNA-binding proteins and unstable plaque-specific RAS in atopic dermatitis development. **(A)** Bar plot showing the top 10 most enriched GO biological process results of specific DE-RBPs co-expressed by specific RAS. The color scale shows the row-scaled significance (-log10 corrected *p*-value) of the terms. **(B)** Sheet exhibiting several key GO biological process results of specific DE-RBPs co-expressed by specific RAS. **(C)** Co-expression analysis of specific DE-RBPs and specific RAS of key GO biological process results. Cutoffs of *p*-value ≤0.01 and Pearson coefficient ≥0.6 or ≤ −0.6 were applied to identify the co-expression pairs. The network shows the co-expressed GO pathway for specific DE-RBPs (the far left part) and specific RAS (the middle left part). The top enriched GO terms of RASGs (the middle right part) are shown in red (the far right part). **(D)** Reads distribution diagram showing clualt3p103059 FYN. Bar plot showing the splicing ratio of clualt3p103059 FYN on the right. Error bars represent mean ± SEM. *: *p*-value ≤0.05, **: *p*-value ≤0.01, and ***: *p*-value ≤0.001.

RBPs refer to a group of highly conserved proteins that have essential functions in regulating gene expression after transcription (Van Nostrand et al., 2020). To further explore the potential regulatory function of our DE-RBPs on RAS, first, we performed the co-expression analysis between specific DE-RBPs and all RAS genes. Subsequently, we conducted a co-expressed GO enrichment analysis for specific DE-RBPs and specific RAS genes (Supplementary Figure S3A). We found that the RAS genes co-expressed with DE-RBPs were mainly enriched in biological pathways related to cytoskeleton organization, inflammatory responses, cellular response to hydrogen peroxide stress, endocytosis, protein stabilization, positive regulation of gene expression, and innate immune response. Notably, the biological pathways related to cytoskeleton organization, inflammatory response, and innate immune response deserve particular attention (Figure 3A). Additionally, we presented tables to display these RAS genes related to cytoskeleton, inflammation, and immunity (Figure 3B). Subsequently, we analyzed and constructed the interaction network between RBPs and RAS genes related to cytoskeleton, inflammation, and immunity (Figure 3C) and found that a large number of RBPs were involved in regulating AS of immune- and cytoskeleton-related genes in AD. Furthermore, we presented the reads distribution maps for the genes *FYN* (Figure 3D) and *ADGRE5* (Supplementary Figure S3B) that underwent AS. These results imply that DE-RBPs may play an important function in the development of AD by regulating the AS of these genes.

### Part 4: Validation of the DE-RBPs and RAS genes in clinical samples

**FIGURE 4:**
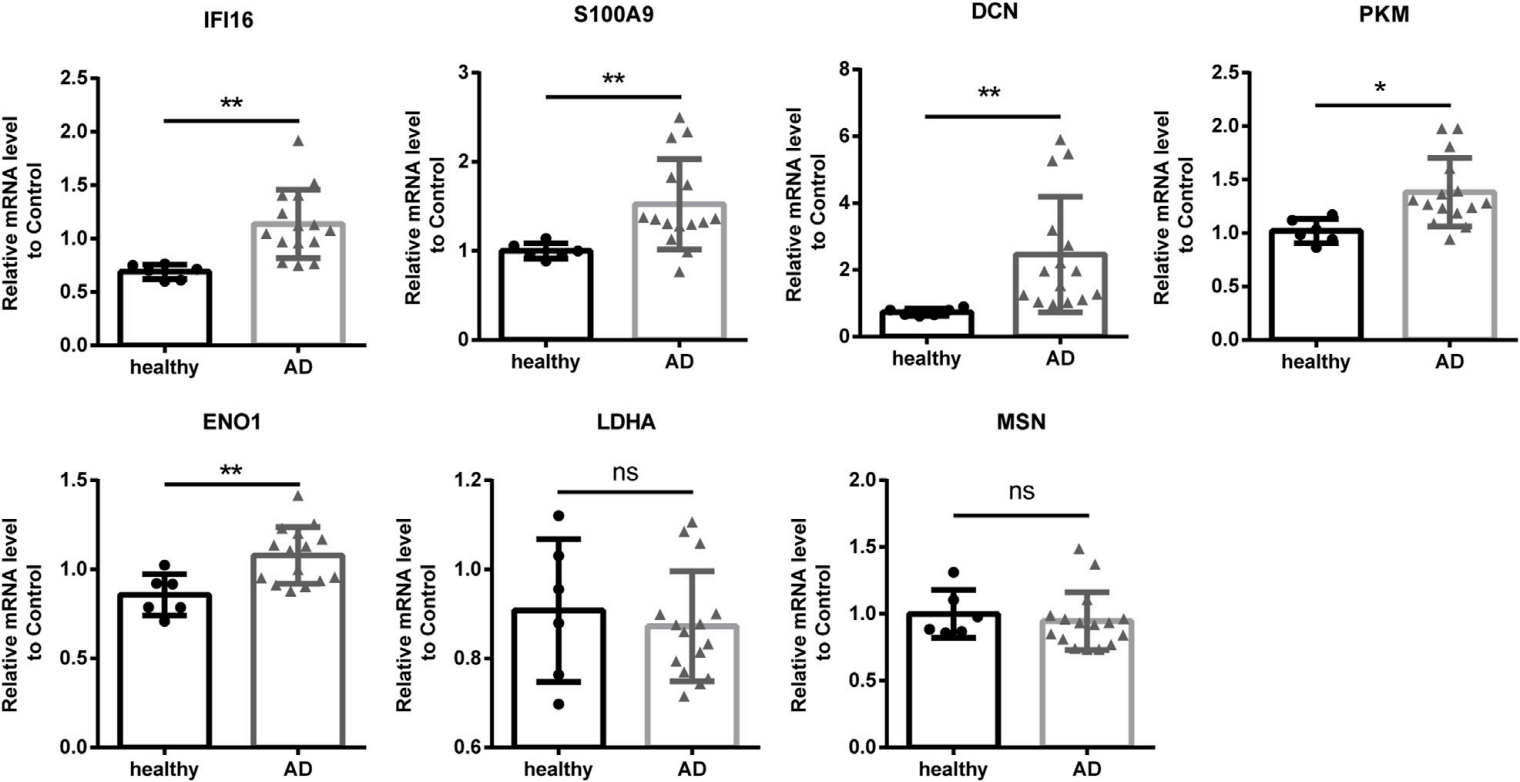
Validation of important RBPs in PBMCs in clinical samples. Results of the qPCR of the gene expression of seven RPBs. RBP, RNA-binding protein; qPCR, quantitative polymerase chain reaction. *: *p*-value ≤0.05; **: *p*-value ≤0.01.

**FIGURE 5:**
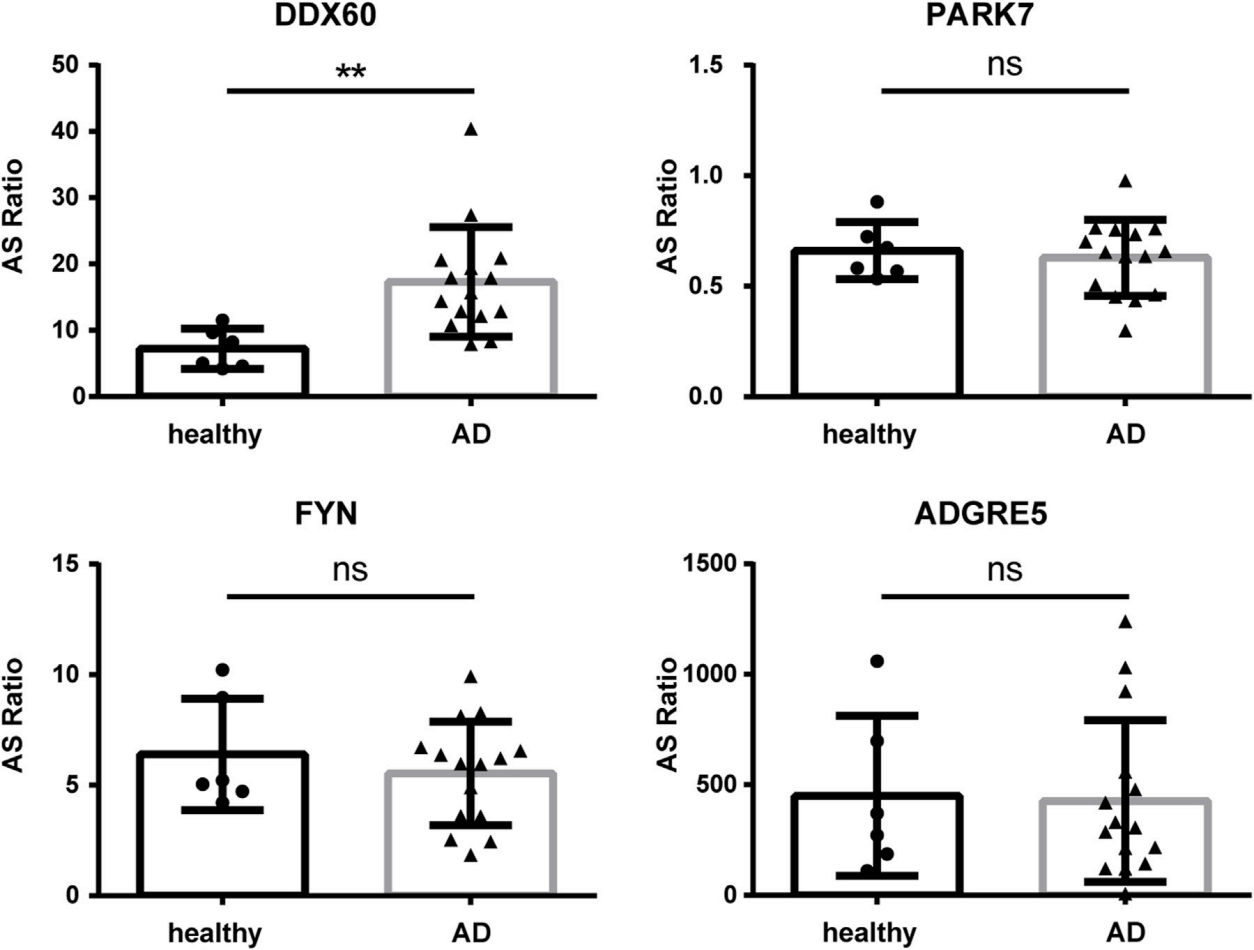
Validation of important RAS genes in PBMCs in clinical samples. qPCR was performed to detect the splicing ratio of four RAS genes. RAS, regulatory alternative splicing; qPCR, quantitative polymerase chain reaction. **: *p*-value ≤0.01.

To further investigate the function and mechanism of DE-RBPs in AD, we selected seven genes that are commonly found on three pathways (as shown in Figure 3C) that are enriched in the co-expression network of DE-RBPs and RAS. Subsequently, we detected their expression in PBMCs of both AD patients and healthy individuals. The expression levels of gene IFI16, S100A9, decorin (DCN), pyruvate kinase M1/2 (PKM), and enolase 1 (ENO1) in PBMCs were significantly upregulated in the AD group compared to the healthy group. However, the expression levels of genes lactate dehydrogenase A (LDHA) and moesin (MSN) in PBMCs showed no significant difference between the AD group and the healthy group (Figure 4). These results suggest that the observed cellular level changes in the above five RBP genes may contribute to the regulation of AS in downstream AD development. Additionally, we selected four RAS genes regulated by RBPs that were related to the immune pathway and then detected their RASEs in PBMCs from both AD patients and healthy individuals. The results showed an increased percentage of RASEs in the gene *DDX60* in AD (Figure 5). This finding suggests that the AS of DDX60 may be regulated by RBP involved in the immune response to AD.

## Discussion

In this study, first, we collected 2141 RBPs from four previous reports (Castello et al., 2012; Gerstberger et al., 2014; Castello et al., 2016; Hentze et al., 2018) and then analyzed the DE-RBPs in AD and healthy skin using the RNA-seq data of AD (GSE121212). To further investigate the potential regulatory role of AD-associated DE-RBPs on RASEs, we performed AS analysis on the above transcriptomic data using the newly published AS analysis software SUVA (v 2.0). Through this analysis, we identified highly conserved RASEs associated with AD. Additionally, we constructed a co-variation network of DE-RBPs and these RASEs, revealing the potential regulatory function of DE-RBPs in the development of AD through the modulation of RASEs. We mainly focused on the biological pathways related to cytoskeleton organization, inflammatory response, and immune response from the top 10 enriched GO biological processes. Subsequently, we selected seven genes that are commonly involved in these three pathways to assess their expression levels in PBMCs from AD patients. We found that IFI16, S100A9, DCN, PKM, and ENO1 were upregulated in PBMCs from AD patients, which is highly consistent with the DE-RBP gene analysis, except DCN. This finding suggests that the expression of these four RBP genes (IFI16, S100A9, PKM, and ENO1) may affect the occurrence and development of AD by regulating AS in biological pathways related to cytoskeleton organization, inflammatory response, and immune response. Finally, we selected four RAS genes regulated by RBPs that were related to the immune pathway to detect their RASEs in PBMCs from AD patients. Remarkably, we found an increased percentage of RASEs in the gene *DDX60*, which is highly consistent with the AS analysis. This finding suggests that the AS of DDX60 may be regulated by RBP involved in the immune response to AD. In conclusion, our findings provide preliminary evidence suggesting that RBPs may influence the development of AD by regulating AS. This offers a potential target for the prevention and treatment of AD.

Immunological aspects, such as the role of the epidermal barrier and abnormal cytokine expressions, are frequently studied in the pathogenesis of AD (Das et al., 2022). In AD, the damaged epidermal barrier facilitates the penetration of allergens, pathogens, and other external irritants into the body and activates keratinocytes. During the dysregulation of immune responses in AD, activated keratinocytes contribute to the pathogenesis of AD through several biological processes (Chieosilapatham et al., 2021). Tsoi LC et al. analyzed RNA-seq data (GSE121212) and observed that AD is characterized by an IL-13-dominant disease phenotype. Additionally, they found that 81% of the dysregulated genes in lesional AD skin are shared with psoriasis (Tsoi et al., 2019). Furthermore, genes involved in skin barrier repair, keratinocyte proliferation, wound healing, and negative regulation of T-cell activation exhibited significant dysregulation in the chronic *versus* acute comparison (Tsoi et al., 2020). In our study, we observed that upregulated genes in the AD LS and AD NS groups were mainly enriched in immune and cytokine signaling pathways, whereas upregulated genes in the AD NS and healthy groups were mainly associated with skin barrier maintenance and repair. These findings suggest that the DEGs either cytokines or RBPs play important roles in the development of AD. Immune response, cell signaling, and epidermal repair are pathways that merit further investigation. Merleev et al. (2022) analyzed RNA-seq datasets including GSE121212, and they found that PCSK9 is a psoriasis susceptibility locus that is negatively related to IL36G; this result established a putative link between PCSK9 and inflammatory cytokine expression (Merleev et al., 2022). Furthermore, PCSK9 has been reported to be associated with inflammation in AD (Valenzuela et al., 2020). In our study, we identified PCSK9 as a DE-RBP gene related to immune and apoptosis pathways in AD. These findings suggest that the role of PCSK9 in AD skin inflammation deserves further investigation.

Cao et al. (2016) found that increased IFI16 expression leads to the development of psoriasis by regulating chemokine production in keratinocytes (Cao et al., 2016). De Andrea et al. (2020) found that IFI16 was detectable in the serum and synovial fluid of psoriatic arthritis patients, especially in cases of elevated C-reactive protein (De Andrea et al., 2020). Dolcino et al. (2012) found that IFI16 regulates local inflammatory responses in dermatitis herpetiformis skin lesions (Dolcino et al., 2012). Yatagai et al. (2013) found that the single-nucleotide polymorphisms of IFI16 might be one of the candidate regions that are associated with the level of total serum IgE (Yatagai et al., 2013). These findings suggest that IFI16 may be involved in the inflammatory response of skin lesions, and therefore, the role of IFI16 in AD skin inflammation deserves further investigation. Research has shown that the levels of S100A9 are elevated in the affected skin and serum of AD patients, and these levels correlate with the severity of AD. S100A9 may play a role in the induction of AD through IL-17A and house dust mites, as well as in inflammation mediated by damage-associated molecular pattern (DAMP) molecules (Jin et al., 2014). In addition, S100A9 has been found to upregulate the expression levels of IL-6, IL-8, and MCP-1 in human keratinocytes, while downregulating the expression levels of filaggrin and loricrin (Kim et al., 2019). These observations indicate that S100A9 may be an important molecule in the pathogenesis and progression of AD. Tohgasaki et al. (2018 ) found that ENO1 expression in the stratum corneum is elevated in the presence of parakeratosis in AD, leading to the disruption of the cellular tight junction barrier in keratinocytes (Tohgasaki et al., 2018). There were fewer studies about DCN and PKM in AD. However, Seidler et al. (2011) found that DCN plays a role in mediating delayed-type hypersensitivity responses by influencing polymorphonuclear leukocyte attachment to the endothelium (Seidler et al., 2011). Bocian et al. (2013) found that DCN enhances interferon-γ (IFN-γ) signal transduction by activating signal transduction transcriptional activator 1 (STAT-1) in delayed hypersensitivity models (Bocian et al., 2013). In asthmatic mouse models and patients, both CD4^+^ T and CD8^+^ T cells exhibited an upregulated expression of DCN (Chatterjee et al., 2021). These results were consistent with our validation of the DCN in clinical samples, although they do not align with the DE-RBP gene analysis, suggesting that DCN may exhibit differential expression in various tissues of AD patients. PKM might be involved in inducing allergic airway inflammation (Jaiswal et al., 2020; van de Wetering et al., 2020). We speculate that these RBP genes may participate in the regulation of inflammatory response and immune response during the development of AD.

AS is a fundamental process that allows a single gene to produce multiple protein isoforms with distinct functions. In the context of AD, AS has been found to play a crucial role in disease pathogenesis. Studies have demonstrated that ASEs can contribute to the dysregulation of key signaling pathways involved in the immune response and skin barrier function. For instance, researchers identified an AS variant of the *FLG* gene, which has been associated with impaired skin barrier function and increased susceptibility to AD (Esparza-Gordillo et al., 2009; Elias et al., 2019). Abnormal RNA splicing in keratinocytes contributes to the development of inflammatory skin diseases. Ni et al. (2022) found that the RNA helicase DDX5 is downregulated in keratinocytes from the inflammatory skin lesions in patients with AD and psoriasis. They also revealed that IL-17D regulates DDX5 expression, thereby controlling inflammation in keratinocytes during inflammatory skin diseases (Ni et al., 2022). These findings highlight the importance of AS in the development and progression of AD, providing potential targets for therapeutic interventions and personalized treatments.

Pasquali et al. (2019) found that DDX60 is highly expressed in keratinocytes and is mainly associated with innate immunity (Pasquali et al., 2019). Kim et al. (2013) found that PARK7/DJ-1 is diminished in AD, and the impaired activity of DJ-1 can have implications for allergic diseases mediated by mast cells (Kim et al., 2013). Morelli et al. (2021) found that the downregulation of PARK7/DJ-1 indicates impairment in innate immune and antioxidant mechanisms in AD (Morelli et al., 2021). Previous reports have indicated that caffeine-proline-histidine amide inhibited FYN and alleviated AD-like phenotypes by inhibiting NF-κB activation (Jeong et al., 2020). Additionally, the anticancer candidate CYC116 inhibits type I hypersensitive immune responses by targeting the expression of FYN kinase in mast cells (Park et al., 2019). CD97/ADGRE5 has been demonstrated to modulate immunity by upregulating macrophage PPAR-γ to inhibit NF-κB activation induced by LPS (Wang et al., 2016). These findings suggest that RBP may play an important role in the development of AD by regulating the AS of these genes.

It is increasingly recognized that AD is a systemic rather than a localized disease and should, therefore, be evaluated from a systemic pathophysiological perspective. Research studies have shown that some certain genes are expressed consistently in both PBMCs and damaged skin in AD (Jujo et al., 1992; Leung, 2000; Hu et al., 2020; Nousbeck et al., 2022; Sekita et al., 2023), and the abnormal expression of these genes is associated with the development of AD (Yu et al., 2020; Nousbeck et al., 2022; Sekita et al., 2023). The well-known features of AD include an increase in Th2 cell cytokines (such as IL-4 and IL-13) and IgE production (Leung, 2000) but a decrease in the production of Th1 cell cytokine IFN-γ in peripheral blood and acute skin lesions (Jujo et al., 1992). Hu et al. (2020) found that the aryl hydrocarbon receptor (AhR) and its downstream regulators are highly expressed in the serum, PBMCs, and skin of AD patients and its correlation with disease severity (Hu et al., 2020). Nousbeck et al. (2022) analyzed the PBMC gene profile of infants with AD, revealing the molecular mechanisms and potential diagnostic markers of pediatric AD (Nousbeck et al., 2022). In this dataset, S100A9, IFI16, and DDX60 were also identified as DEGs, consistent with our analysis results. In addition, considering the limited availability of patient specimens in our allergy department, the easiest to obtain is blood. Moreover, we have readily available PBMC samples in our biobank. For the convenience of subsequent research, we chose to validate in PBMCs.

However, our study has certain limitations, including a small sample size for clinical validation, the lack of grading for skin lesion severity in patients, potential differences in RBP and AS between PBMCs and skin, the requirement for additional validation of RBP and RASE genes, and the necessity to study the function of RBP and RASE genes at the cellular and animal levels. Nonetheless, our findings are innovative as they uncover genes (IFI16, S100A9, PKM, and ENO1) associated with cytoskeleton organization, inflammatory response, and immune response. These genes potentially affect the occurrence and development of AD by regulating AS, including the regulation of DDX60 in biological pathways. In conclusion, this study provides additional evidence to support the involvement of DE-RBPs in AD and highlights the potential functional roles of these RBPs in regulating AS and downstream gene expression.

## Data Availability

The original contributions presented in the study are included in the article/Supplementary Material; further inquiries can be directed to the corresponding author.
