# Supplementary material for: Genome-wide identification of dysregulated alternative splicing and RNA-binding proteins involved in atopic dermatitis
Source: Front Genet. 2024 Mar 1;15:1287111. doi: 10.3389/fgene.2024.1287111 (PMC10940350; doi:10.3389/fgene.2024.1287111)
Supplement: Supplementary file 1 [file DataSheet1.PDF]

Table S1 Demographic data of samples from RNA-seq data (GSE121212)

Trait	ID	Sex	Age (year)	Severity (ScorAD)	Rhinitis	Asthma	biopsy site non-lesional	biopsy site lesional
AD	AD1	female	48	35.5	yes	yes	upper arm flexural right	upper arm flexural left
AD	AD2	male	43	44.5	yes	yes	upper arm flexural left	upper arm flexural right
AD	AD3	male	25	25	no	no	upper arm flexural left	upper arm flexural right
AD	AD4	male	33	15	yes	no	upper arm flexural left	antecubital fossa left
AD	AD5	male	45	32	yes	yes	upper arm flexural left	antecubital fossa left
AD	AD6	male	45	36	yes	yes	upper arm flexural left	upper arm flexural left
AD	AD7	male	37	48	yes	no	upper	upper arm

							arm flexural left	flexural left
AD	AD8	female	25	18.5	no	no	upper arm flexural left	upper arm flexural left
AD	AD9	female	42	38	yes	yes	upper arm flexural left	upper arm flexural left
AD	AD10	female	22	24.5	no	no	upper arm flexural left	antecubital fossa left
Healthy	Ctrl1	female	42		no	no	upper arm flexural left	
Healthy	Ctrl2	female	25		no	no	upper arm flexural left	
Healthy	Ctrl3	female	28		no	no	upper arm flexural left	
Healthy	Ctrl4	female	24		no	no	upper arm	

							flexural left	
Healthy	Ctrl5	male	28		no	no	upper arm flexural right	
Healthy	Ctrl6	female	33		no	no	upper arm flexural left	
Healthy	Ctrl7	female	27		no	no	upper arm flexural left	
Healthy	Ctrl8	male	26		no	no	upper arm flexural left	
Healthy	Ctrl9	male	42		no	no	upper arm extensor right	
Healthy	Ctrl10	male	24		no	no	upper arm flexural left	

Table S2 Demographic data of samples from PBMCs database

Trait	ID	Sex	Age (year)	Severity (ScorAD)	Rhinitis	Asthma	Allergen	TIgE (ku/l)
AD	AD1	female	61	20	no	no	nickel sulfate and textile dye mix	13.1
AD	AD2	female	34	42	yes	yes	dust mites and *Platanus* pollen	538
AD	AD3	male	32	48	no	no		81.3
AD	AD4	female	42	34.5	no	no		24
AD	AD5	female	24	35	yes	no		150
AD	AD6	male	44	23	no	no	dust mites	63.2
AD	AD7	female	54	26	no	no		103
AD	AD8	female	22	49	no	no	dust mites	798
AD	AD9	female	22	23	no	no		17.5
AD	AD10	male	35	21.5	yes	no	dust mites	57.4
AD	AD11	male	29	33.5	no	no		69.6
AD	AD12	female	42	42	no	no		3.3
AD	AD13	male	37	21	yes	no		8.44
AD	AD14	female	36	20	no	no		140
AD	AD15	male	25	18	yes	no	cockroach	437
healthy	Ctrl1	male	31		no	no		108
healthy	Ctrl2	female	22		no	no		174
healthy	Ctrl3	male	37		no	no		8.9
healthy	Ctrl4	male	29		no	no		46
healthy	Ctrl5	female	23		no	no		121
healthy	Ctrl6	male	24		no	no		278

Table S3

Gene	Primer
IFI16	F: AGACTGAAGACTGAACCTGAAGA<br>R: GAACCCATTGCGGCAAACATA
S100A9	F: GGTCATAGAACACATCATGGAGG<br>R: GGCCTGGCTTATGGTGGTG
DCN	F: ATGAAGGCCACTATCATCCTCC<br>R: GTCGCGGTCATCAGGAACTT
PKM	F: ATGTCGAAGCCCCATAGTGAA<br>R: TGGGTGGTGAATCAATGTCCA
ENO1	F: AAAGCTGGTGCCGTTGAGAA<br>R: GGTTGTGGTAAACCTCTGCTC
LDHA	F: ATGGCAACTCTAAAGGATCAGC<br>R: CCAACCCCAACAACTGTAATCT
MSN	F: ATGCCCAAAACGATCAGTGTG<br>R: ACTTGGCACGGAACTTAAAGAG
DDX60	M/AS-F: GCTGCTGCCTGTGCCTCCAA<br>AS-R: TGAGTCCGCGGACTGCCATTT<br>M-R: AGAAGCACAGGACTGCCATTT
PARK7	M-F: CTGGTGTGGGGCTTGTAAACA<br>AS-F: CGGGGTGCAGGCTTGTAAACA<br>M/AS-R: TCTCCATTTCCTCTGCTCCT
FYN	M-F: ATCAGCTTTCTCTGAGTAATGT<br>AS-F: TGCAGCTCTCTCTGAGTAATGT<br>M/AS-R: TTGGGATGATATGAAAGGAGAC
ADGRE5	M/AS-F: GGGATATGAGCCTGTTTCTG<br>AS-R: TCGTCCACATCTTGACAGGT<br>M-R: GGCAGCTGTACTTGACAGGT

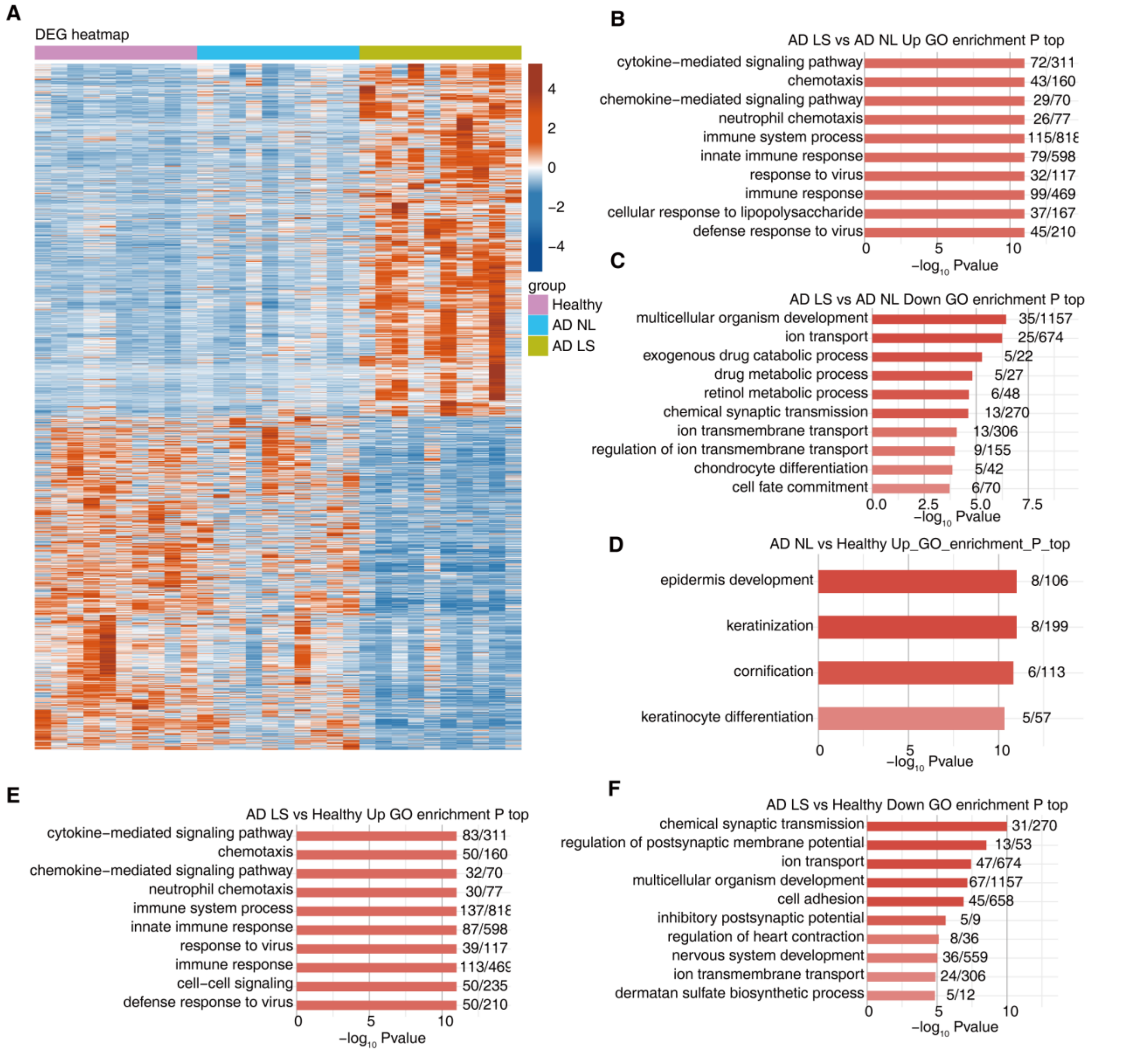

Figure S1. Transcriptome analysis of differentially expressed RBP genes (DE-RBPs) in atopic dermatitis lesional and atopic dermatitis non-lesional samples compared with healthy samples.

A. The heatmap diagram showing the expression profile of DEG.

B-C. The top 10 most enriched GO terms (biological process) were illustrated for overlap up-regulated/down-regulated DEGs in the AD_LS vs. AD_NL groups.

D. The most enriched GO terms (biological process) were illustrated for overlap up-regulated DEGs in the AD_NL vs. Healthy groups.

E-F. The top 10 most enriched GO terms (biological process) were illustrated for overlap up-regulated/down-regulated DEGs in the AD_LS vs. Healthy groups.

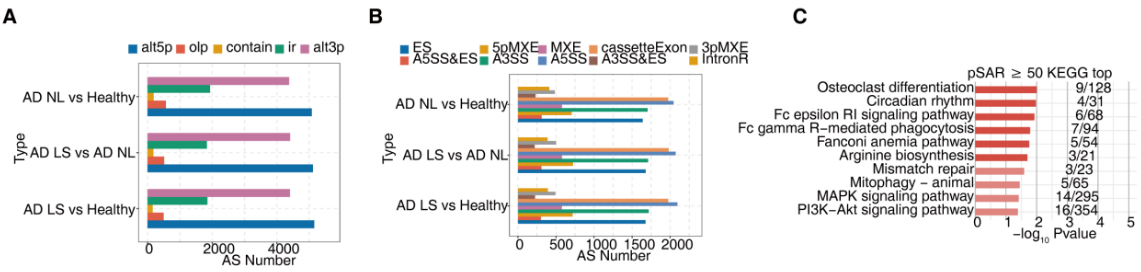

Figure S2. A large number of alternative splicing events related to inflammatory and immune responses were identified in atopic dermatitis development.

A. Bar plot showing the number of regulatory AS detected by SUVA in each group.

B. Splice junction constituting AS events detected by SUVA was annotated to classical AS event types. The number of each classical AS event type was shown with a bar plot.

C. The top 10 most enriched KEGG terms were illustrated for specific RAS in the three groups.

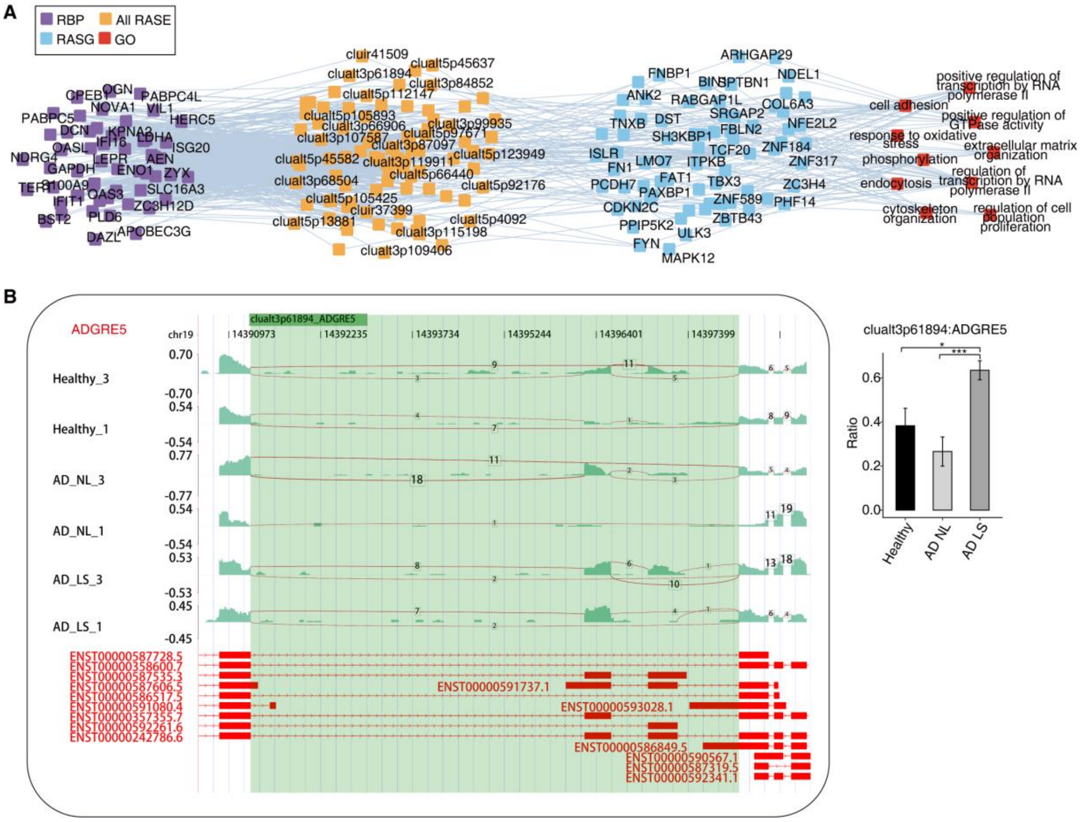

Figure S3. Construction of co-disturbed network between RNA binding protein and unstable plaques specific RAS in atopic dermatitis development.

A. Co-expression analysis of specific DERBP and All RAS. Cutoffs of P value ≤ 0.01 and Pearson coefficient ≥ 0.6 or ≤ -0.6 were applied to identify the co-expression pairs. The network shows the co-expressed GO pathway for specific DERBP and specific RAS.

B. Reads distribution diagram showing clualt3p61894 ADGRE5. Bar plot showing the splicing ratio of clualt3p61894 ADGRE5 on the right. Error bars represent mean ± SEM. *: P value ≤ 0.05, **: P value ≤ 0.01,***: P value ≤ 0.001.
